# Delayed Neurological Deficits Caused by Epidural Leakage of Intravertebral Contents in a Patient With Osteoporotic Vertebral Fracture

**DOI:** 10.7759/cureus.100441

**Published:** 2025-12-30

**Authors:** Fukuda Mamoru, Yuma Hiratsuka, Michiru Katayama, Yasufumi Ohtake

**Affiliations:** 1 Neurological Surgery, Nakamura Memorial Hospital, Sapporo, JPN

**Keywords:** delayed neurological deficit, osteoporosis, osteoporotic vertebral fracture, thoracic spine, vertebral osteonecrosis, vertebroplasty

## Abstract

Delayed neurological deficits (DND) after osteoporotic vertebral fractures (OVF) are a rare but serious complication. In patients with Parkinson's disease (PD), involuntary movements such as dyskinesia may increase biomechanical stress, potentially exacerbating spinal instability. We report the case of a 74-year-old woman with PD and a history of T12 OVF, who presented with progressive bilateral thigh pain and gait disturbance despite conservative treatment and bed rest. Imaging revealed a posterior epidural mass at T12 with an atypically high CT attenuation (300-340 hu), inconsistent with hematoma. MRI demonstrated a fluid level within the T12 vertebral body, suggesting vertebral instability and possible content extrusion. Emergency surgery, including vertebroplasty, posterior instrumentation, and decompressive laminectomy, was performed. The epidural lesion was pathologically identified as extruded vertebral contents, including hematopoietic marrow, fatty tissue, bone fragments, and necrotic debris, with no evidence of hematoma, inflammation, or malignancy. The patient experienced rapid postoperative improvement and was discharged ambulatory. She remained neurologically intact at the 18-month follow-up. This case highlights a rare mechanism of DND in OVF: epidural migration of vertebral contents through posterior wall failure, where dyskinesia may have been a contributing factor. Such lesions may mimic hematomas but show atypical imaging characteristics. Awareness of this pathology, along with timely surgical intervention and pathological confirmation, is crucial for accurate diagnosis and favorable outcomes in similar patients.

## Introduction

Delayed neurological deficits (DND) are a well-recognized consequence of osteoporotic vertebral fractures (OVF) [1]. A significant complication of OVF is Kummell disease, which typically involves a delayed collapse of a vertebra following trauma, associated with avascular necrosis and the possible formation of an intravertebral vacuum cleft. Conservative management, including osteoporosis treatment, is crucial for Kummell disease, and timely surgical procedures like vertebral augmentation might be required to prevent neurological problems [2]. While uncommon, DND can also be caused by epidural hematomas that form after minor, repeated injuries; osteoporotic vertebral collapse, in particular, may result in epidural hematoma [3]. Additionally, research indicates that individuals with Parkinson's disease (PD) face a considerably greater risk of OVF than those without the disease [4]. This report describes a case of DND caused by epidural leakage of intravertebral contents, including bone fragments and fatty marrow, in a patient with PD and dyskinesia.

## Case presentation

**Figure 1:**
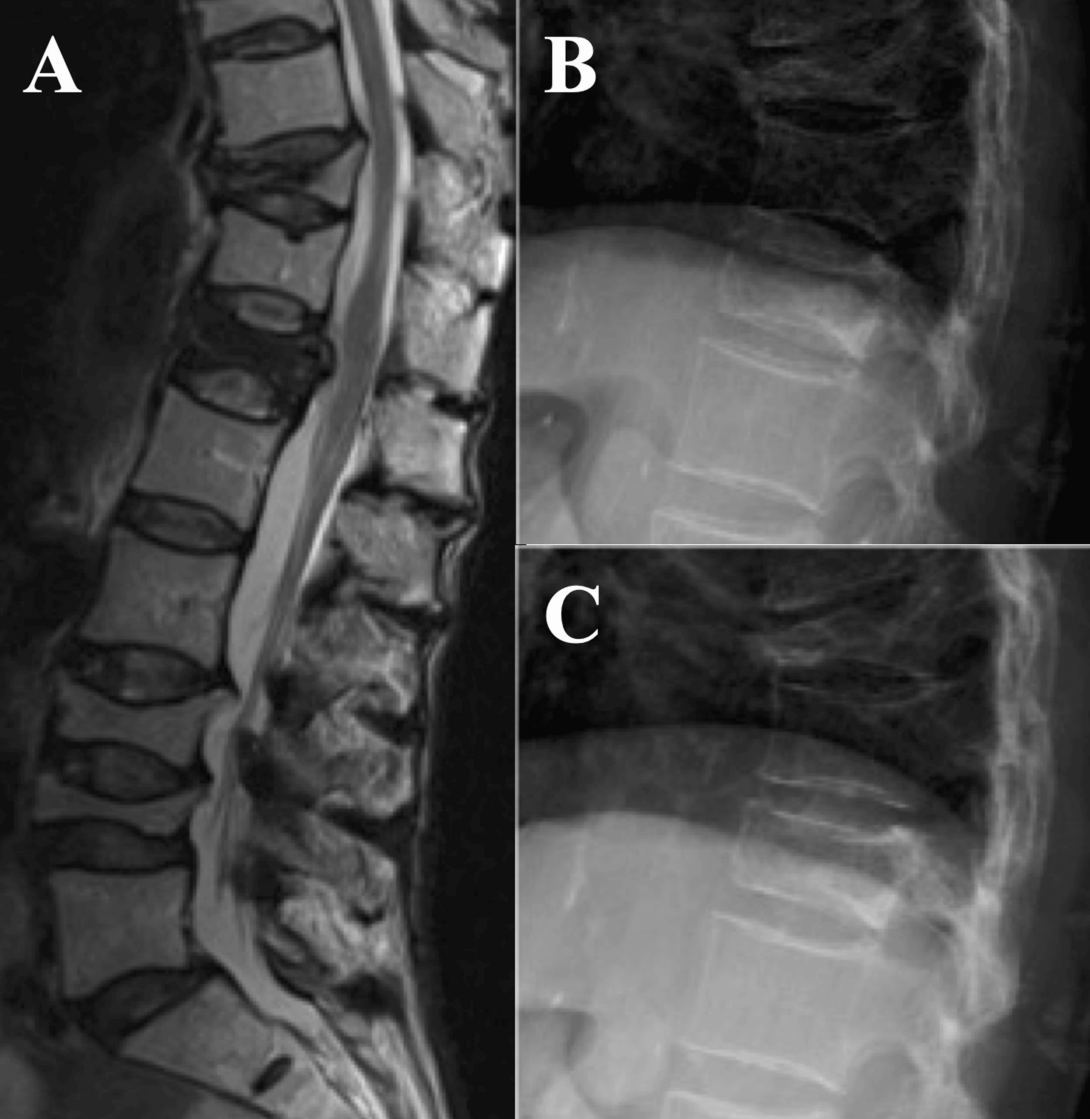
Imaging findings of the thoracic and lumbar vertebrae A: Sagittal T2-weighted MRI on admission showing T12 vertebral fracture and T10, L3, and L4 chronic OVF. B: Standing X-ray. C: Supine X-ray showing slight intravertebral instability. OVF, osteoporotic vertebral fractures

A 74-year-old woman with PD and OVF, who had been receiving denosumab therapy for osteoporosis, presented to our hospital with bilateral lateral thigh pain and difficulty walking. Her back pain started six months earlier, leading to an OVF diagnosis at an orthopedic clinic where she received conservative treatment with an external brace. Four months later, her symptoms worsened without recent trauma. On examination, she reported bilateral lateral thigh pain (Numeric Rating Scale (NRS) 6-7/10) and showed weakness in the tibialis anterior muscle (manual muscle testing (MMT) 3/5). Deep tendon reflexes were normal with a negative Babinski sign and straight leg raise test. Her PD was at Hoehn and Yahr stage IV (Movement Disorder Society-Unified Parkinson's Disease Rating Scale (MDS-UPDRS) score 9) with notable dyskinesia affecting the trunk and limbs (Abnormal Involuntary Movement Scale total score 14). Lumbar MRI in Figure 1A showed a T12 vertebral fracture and chronic OVF at T10, L3, and L4 levels. X-rays in Figure 1B and Figure 1C indicated mild intravertebral instability, and bone densitometry confirmed osteoporosis (T-score -2.9 SD).

**Figure 2:**
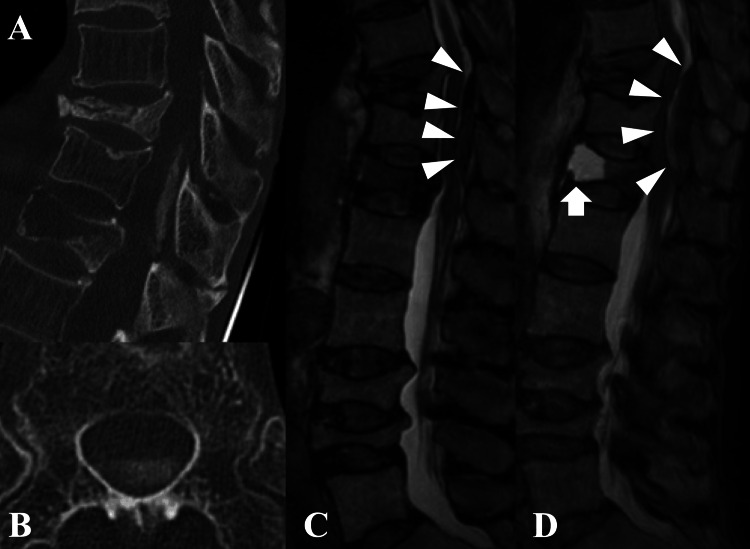
Thoracic CT and MRI A: Sagittal CT showing a space-occupying lesion on the dorsal epidural surface of T12. B: Axial CT showing a space-occupying lesion on the dorsal epidural surface of T12. C: Sagittal T2-weighted MRI revealed an epidural lesion (arrowheads). D: Sagittal T2-weighted MRI when paralysis and sensory disturbance worsened. The image confirmed the enlarging epidural lesion (arrowheads) and demonstrated niveau formation within the T12 vertebral body (arrow).

The patient was admitted for observation as the cause of her neurological symptoms remained unclear. A CT scan after admission revealed a space-occupying lesion on the posterior epidural surface of T12. As her symptoms were stable, we continued observation. Days later, her paralysis and sensory disturbance worsened. Follow-up CT (Figure 2A and Figure 2B) showed an expanded epidural lesion with a CT value of 300-340 HU, which was unusual for a hematoma or abscess. MRI (Figure 2C and Figure 2D) confirmed the enlarging lesion and showed niveau formation within the T12 vertebral body.

**Figure 3:**
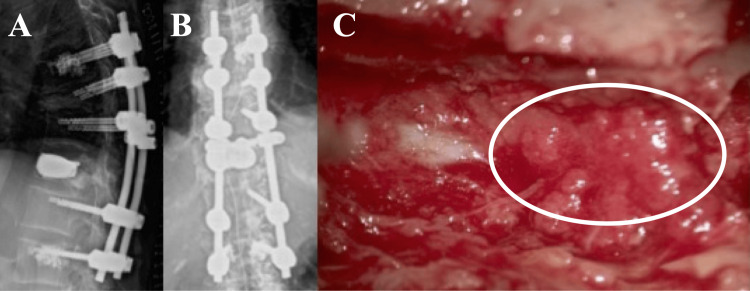
Surgical findings We performed T12 vertebroplasty and stenting, T9-L2 posterior fusion, and T10-12 laminectomy to remove the epidural mass. The epidural mass appeared as a soft, ragged, yellowish lesion, as indicated by the circle.

Due to progressive symptoms, emergency surgery was performed, including T12 vertebroplasty and stenting, posterior fusion from T9 to L2, and laminectomy from T10 to T12 to remove the epidural mass. The lesion appeared as a soft, ragged, yellowish mass (Figure 3).

**Figure 4:**
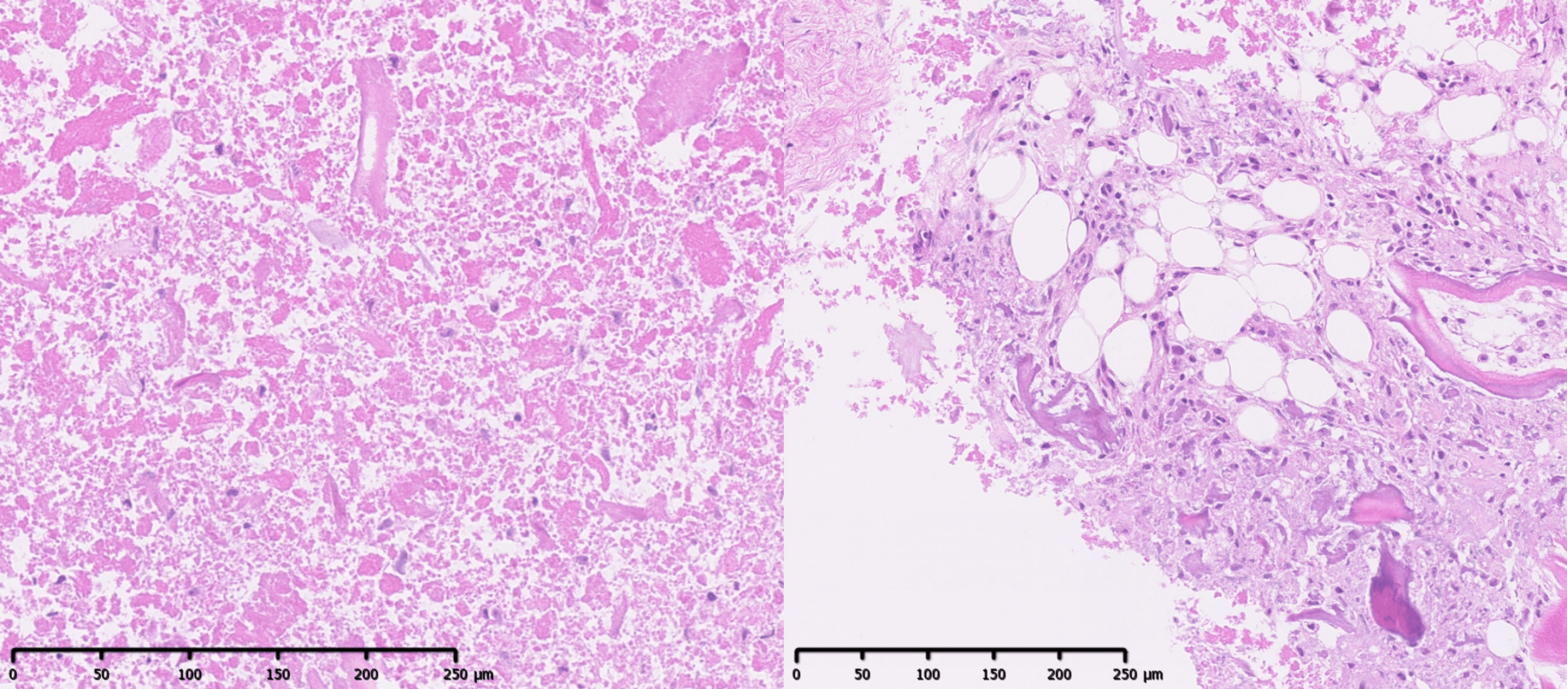
Histopathological findings Hematoxylin and eosin staining of the removed space-occupying lesion revealed hematopoietic marrow, fatty medulla, bone fragments, and necrotic material. There was no evidence of inflammatory cell infiltration or malignancy.

Histopathological examination (Figure 4) revealed hematopoietic marrow, fatty medulla, bone fragments, and necrotic material without inflammatory cells or malignancy.

These findings suggested vertebral body fluid had extruded into the spinal canal through the posterior wall fracture. Postoperatively, the patient's paralysis improved rapidly. After rehabilitation, she was discharged walking independently. At 18-month follow-up, she maintained good clinical status with no new fractures, neurological symptoms, or implant-related issues.

## Discussion

This case report highlights two main findings, particularly significant given that DND after OVF can occur through various mechanisms, including compression from a space-occupying lesion such as an epidural hematoma [1]. First, our case demonstrates that dyskinesia in a patient with PD and OVF can potentially cause the epidural leakage of intravertebral contents, leading to DND even while the patient is on bed rest. Second, the epidural lesion, composed of OVF debris (hematopoietic marrow, fatty medulla, bone fragments, and necrotic material), presented with atypical CT values (approximately 340 hu), differentiating it from a typical hematoma and thus complicating the initial diagnosis. These findings underscore a unique pathway for DND in OVF patients who also have PD and emphasize the diagnostic challenges that such specific presentations can pose.

Our first key finding is the occurrence of epidural leakage of vertebral contents leading to DND in a patient with both PD and OVF. DND following OVF, first described by Kempinsky et al. [5], can arise from several causes; these include nerve compression by bone fragments, kyphotic deformity, instability at the fracture site, or direct compression by space-occupying lesions like epidural hematomas [1]. While direct compression by such space-occupying lesions has been reported in a few cases [6-8], these are often associated with minor trauma [6]. In our case, DND developed despite strict bed rest in a patient with severe dyskinesia, raising the question of whether repetitive involuntary movements might have contributed to posterior wall failure and debris extrusion. However, the temporal relationship between dyskinesia and the development of DND could not be clearly established in this case, and osteoporotic fractures commonly progress regardless of mechanical stress. Therefore, while dyskinesia may represent a potential contributing factor, we cannot definitively establish it as the primary cause of epidural accumulation of vertebral contents in this patient.

The posterior location of extruded vertebral contents in this case may be related to anatomical characteristics of the epidural space. The anterior epidural space is relatively narrow and densely occupied by venous structures, with meningovertebral ligaments forming strong anatomical adhesions between the vertebral body, posterior longitudinal ligament, and dura, which may limit expansion capacity and serve as a barrier to anterior accumulation [9-11]. In contrast, the posterior epidural space appears to be larger and contains compressible adipose tissue with less rigid attachments, bounded by the elastic ligamentum flavum [9,10]. Dorsal meningovertebral ligaments connecting the dura to the ligamentum flavum also exist; the spaces between these ligaments are filled with loose areolar connective tissue and fat [12]. The occurrence of these dorsal ligaments increases caudally in the lumbar spine, being lower at upper lumbar levels (20% at L1) compared to lower levels (97% at L5-S1) [12]. Although data on the thoracic spine are limited, the lower thoracic location in our case might be associated with a similarly reduced occurrence of dorsal meningovertebral ligaments. When posterior vertebral wall failure occurs in a patient on prolonged bed rest, extruded material might preferentially migrate toward the posterior epidural space, following both gravitational force in the supine position and the path of lower resistance. These anatomical differences could potentially contribute to the posterior accumulation of vertebral contents observed in our patient, although other factors may also be involved.

Our second key finding concerned the nature of the epidural space-occupying lesion, specifically that it was OVF debris, and its atypical CT value. While an epidural hematoma can be associated with osteoporotic vertebral collapse and cause neurological deficits [3], the lesion in our patient was different. The CT value of the epidural lesion was approximately 340 HU, suggesting a composition more complex than a simple fluid collection or fresh hematoma, which typically would have a lower hu value (e.g., hematomas around 80 HU) [13]. Pathological examination confirmed this, revealing hematopoietic marrow, fatty medulla, bone fragments, and necrotic material, with no evidence of hematoma. These components are highly consistent with the contents expected from an osteonecrotic vertebral body; indeed, histopathological studies of vertebral osteonecrosis describe features such as dispersed necrotic bone fragments and altered marrow, including marrow fat necrosis or its replacement by fibrous tissue [14]. Supporting the osteonecrotic origin of this debris, radiological findings within the affected T12 vertebra in our case included an MRI fluid sign (observed as a fluid level). Both the MRI fluid sign and the intravertebral vacuum phenomenon (cleft) are recognized radiological indicators of vertebral osteonecrosis [13,14], with the fluid sign reliably correlating with osteonecrosis [14] and the vacuum phenomenon being a specific sign of this condition [15]. The extruded material forming the epidural lesion in our patient thus likely originated from such an osteonecrotic vertebra exhibiting these radiological and pathological characteristics. Therefore, pathological examination remains crucial for an accurate diagnosis and to guide appropriate management, especially to differentiate the lesion from other possibilities such as an abscess, tumor, or a typical hematoma.

## Conclusions

We reported a rare case of DND in a patient with both OVF and PD. The cause was an epidural lesion composed of intravertebral debris, not a typical hematoma, which presented with an unusually high CT value. Dyskinesia was considered a potential contributing factor to the epidural leakage. This rare etiology may warrant consideration when DND occurs in patients with both OVF and PD.
